# Tumor Microenvironment in Thymic Epithelial Tumors: A Narrative Review

**DOI:** 10.3390/cancers14246082

**Published:** 2022-12-10

**Authors:** Apostolos C. Agrafiotis, Vasiliki Siozopoulou, Jeroen M. H. Hendriks, Patrick Pauwels, Senada Koljenovic, Paul E. Van Schil

**Affiliations:** 1 Department of Thoracic and Vascular Surgery, Antwerp University Hospital, University of Antwerp, B-2650 Edegem, Belgium; 2 Laboratory of Pathology, Antwerp University Hospital, University of Antwerp, B-2650 Edegem, Belgium

**Keywords:** thymus, tumor microenvironment, thymoma, thymic carcinoma, immunotherapy

## Abstract

**Simple Summary:**

The tumor microenvironment (TME) is defined as the environment surrounding a tumor. There is a close, dynamic relation and interaction between the tumor and its neighboring microenvironment. There are some particularities of the thymus itself and of the TME of thymic epithelial tumors that hinder the routine use of targeted therapies, including immune checkpoint inhibitors. The understanding of the unique characteristics of the TME of thymic epithelial tumors could possibly result in the development of novel promising therapies.

**Abstract:**

The tumor microenvironment (TME) is a complex and constantly changing entity. The TME consists of stromal cells, fibroblasts, endothelial cells, and innate and adaptive immune cells. Cancer development and progression occurs through this interplay between the tumor and the adjacent stroma. Cancer cells are capable of modifying their microenvironment by secreting various message-carrying molecules, such as cytokines, chemokines, and other factors. This action causes a reprogramming of the neighboring cells, which are enabled to play a crucial role in tumor survival and progression. The study of TME has many clinical implications in terms of cancer therapeutics because many new drugs, such as antibodies, kinase inhibitors, and liposome formulations that can encapsulate anti-cancer drugs, can be developed. Although chemotherapy is considered the standard of treatment for advanced disease, recent research has brought to light immunotherapy as a possible systemic alternative. However, the complex structure and function of the thymus hinders its routine use in clinical practice. The aim of this review paper is to discuss the recent advances in the investigation of the unique characteristics of the TME of thymic epithelial tumors that could possibly lead to the development of novel promising therapies.

## 1. Introduction

The tumor microenvironment (TME) is defined as the environment surrounding a tumor. It includes the vascular endothelial cells, innate and adaptive immune cells, fibroblasts, pericytes, signal-carrying molecules, and the extracellular matrix (ECM) [1,2]. There is a close, dynamic relation and interaction between the tumor and its neighboring microenvironment. This complex interaction plays a central role in cancer development and metastasis [3,4]. This interplay between the tumor and its environment has been observed since the early stages of research on cancer and, consequently, crucial steps in cancer evolution, such as tumor-induced angiogenesis and peripheral immune tolerance, were understood [3]. Similarly, the immune cells surrounding the tumor can impact the evolution of cancer cells [3]. Recently, the TME was brought in the center of interest of many researchers because its study has many clinical implications in terms of cancer therapeutics. The identification and understanding of the different players of the TME could lead to the development of many new drugs, such as antibodies, kinase inhibitors, and liposome formulations that can encapsulate anti-cancer drugs [3,5].

The thymus is an organ of the immune system that is responsible for the development of adaptive immunity [6]. In the thymus, the maturation of thymus cell lymphocytes or T cells, which are important elements of the adaptive immune function, occurs. More specifically, the complicated structure of the thymus offers a unique microscopic environment that guides the maturation of thymocytes and trains T cells to become self-tolerant [6]. Thymic epithelial tumors (TET) are rare neoplasms of the prevascular, or formerly called anterior, mediastinum [7]. They develop from the epithelial thymic cells. This heterogeneous group of tumors consists of thymomas and thymic carcinomas [7]. Thymic carcinomas are less frequent among TET, but they are more aggressive clinically. The variations of histological types have resulted in a recrudescence in research aiming to identify the genetic markers and the oncogenic pathways in the TET that can lead to the development of molecular-targeted therapies [8]. However, the histological heterogeneity that characterizes TET, together with the absence of standard mutations, renders that research difficult [9]. Although chemotherapy is the standard of treatment for advanced disease, recent research has brought to light immunotherapy as a possible systemic alternative [9]. The aim of this review paper is to discuss the recent advances in the investigation of the unique characteristics of the TME of TET that could possibly lead to the development of novel promising therapies.

## 2. Materials and Methods—Search Strategy

This paper was designed according to the recent recommendations on the quality assessment of narrative review articles [10]. PubMed research was conducted using the terms [tumor] AND [microenvironment] AND [thymic epithelial tumors] OR [thymomas]. Papers concerning pediatric cases and non-English literature papers were excluded. As the present study is not a systematic review, the papers were selected according to pertinence. The majority of studies were retrospective cases series and, consequently, papers with a higher level of evidence have not been identified. Among the papers dealing with genetic alterations in thymic tumors, priority was given to recent ones. The references of selected papers were sought in order to find other pertinent articles.

## 3. Results

### 3.1. The Particularities of TME of TET and the Limitations in the Use of Immunotherapy

Surgical resection is considered as the standard treatment for early-stage thymomas, whereas radiation therapy and chemotherapy are reserved for advanced or recurrent tumors and for patients who are not candidates for surgical resection [8,9]. However, as immunotherapy has shown promising results in other solid tumors, it has gained the interest of researchers who investigate its efficacy in TET [11]. Indeed, there are clinical trials that evaluate the use of these agents in the treatment of TET, revealing encouraging preliminary clinical outcomes [12,13]. What is interesting in the case of TET is the key role of the thymus gland in the development of adaptive immune responses, where the maturation of T-cells takes place [9,11]. For that reason, there are severe side-effects (auto-immune toxicity) that have been observed in patients with TET receiving immune checkpoint inhibitors (ICI) [9,11], and they constitute an important obstacle that limit widespread and routine use. Immune-related adverse events generally represent an acceptable morbidity, but, in some cases (ranging from 0.36% to 1.23% determined by the type of ICI used), they can be fatal. Fatal toxicity is less frequent with anti-programmed death 1 (anti-PD-1) receptor and anti-programmed death ligand 1 (anti-PD-L1), followed by anti-Cytotoxic T lymphocyte-associated protein 4 (anti-CTLA-4), and more frequent with their combined administration (anti-PD1/PDL1 and anti-CTLA-4) [14].

There are studies that have identified PD-L1 expression in the majority of TET [15,16]. Padda et al. examined 69 TET and found 68% of cases with PD-L1 overexpression [16]. Katsuya et al. examined a series consisting of 141 TET and discovered high PD-L1 expression in 70% of thymic carcinoma specimens, but only in 23% of thymomas [17]. Similarly, Yokoyama et al. observed increased PD-L1 expression in 80% of thymic carcinoma specimens [18]. However, there is controversy as far as survival is concerned, and, therefore, the prognostic influence and benefit of PD-L1 is debated. Padda et al. demonstrated that increased PD-L1 expression resulted in decreased overall survival (OS), whereas Katsuya et al. described no association between PD-L1 expression and OS [16,17]. Bedekovics et al. found that 69% of thymomas and 43% of thymic carcinomas in their series were highly positive (positive tumor cells ≥50% or positive immune cells ≥10%) [15].

As already mentioned, TET are characterized by histological heterogeneity, and it is observed that most patients are refractory to immunotherapy because of primary or acquired resistance [19,20]. Contrary to standard cytotoxic therapy, the therapeutic action of immunotherapy agents occurs by triggering the anti-tumor immune reaction, which is based on the immunoregulative process that occurs between cancer cells and the TME [19]. Therefore, to help guide the selection of patients who could benefit from this kind of treatment and optimize its outcomes, more specific biomarkers and more pertinent predictive elements for identifying these patients who will potentially respond to these therapeutic modalities are required [19]. Su et al. established an immune-related long noncoding RNAs (IRLs) classifier to accurately identify the response of patients suffering from TET. According to the authors, long non-coding RNAs (LncRNAs) can modulate the immune response by managing the homeostasis, TME, anti-inflammatory factors, and immune cell function [19].

Another issue that needs to be taken into account is the absence of standard actionable mutations, which is the main reason for the lack of effective targeted therapies in TET [18,19]. However, high expression of PD-L1 on tumor cells and abundant CD8+ lymphocytes provide a strong basis for the administration of ICI, which target the PD-1/PD-L1 pathway in the case of TET. Two phase II trials evaluated the efficacy of pembrolizumab (PD-1 inhibitor) in patients with recurrent TET. Giaccone et al. investigated thymic carcinoma cases and reported a response rate of 22.5%. Cho et al. evaluated patients with thymomas and thymic carcinomas, and they reported response rates of 28.6% and 19.2%, respectively. The incidence of adverse events was elevated in thymoma compared to thymic carcinoma (71.4% v 15.4%, respectively) [12,13,21]. Both trials identified an association of high PD-L1 expression with enhanced oncological response, but did not associate PD-L1 expression to adverse events. For those reasons, the development of pertinent biomarkers that could select patients eligible for this treatment is of paramount importance.

### 3.2. The Impact of PD-L1 Expression and the Presence of Different Cell Types in the TME of TET

PD-L1 is an immunomodulatory glycoprotein expressed on antigen-presenting cells. PD-L1 binds to the PD-1 receptor, which is expressed on the surface of T cells, and plays an important role in immune response modulation through the suppression of cytokine production, T-cell proliferation, and T-cell adhesion [11,22,23,24,25,26]. In general, the TME of subtypes A, AB, B1, and B2 thymomas is infiltrated mainly by immature T-cells (CD4+ CD8+), which means that their immune status simulates the normal thymocytes [27]. In the case of advanced WHO histological types (B3 thymomas and thymic carcinomas), there are fully differentiated cytotoxic T-cells. On the contrary, the role of B-cells is largely unknown. They are present in a minority of thymomas, but their role is clearer in the development of autoimmune conditions [28].

Arbour et al. investigated the expression of PD-L1 in the membrane of tumor cells, CD3+ and CD8+ tumor-infiltrating lymphocytes (TILs), co-stimulatory such as CD137, glucocorticoid-induced TNFR-related protein (GITR) and inducible T cell costimulator (ICOS), and co-inhibitory immune checkpoint molecules (PD-1, CTLA-4, and T cell immunoglobulin and mucin-domain-containing protein, TIM-3) in tissue samples from 23 patients with TET (12 thymomas and 11 thymic carcinomas) [11]. PD-L1 positivity (≥25% of tumor membrane expression) was frequent in TET (65%), more common in thymomas compared to thymic carcinomas, and associated with longer OS compared to PD-L1 negative tumors [median OS (mOS) 87 months versus 29 months]. The difference in PD-L1 positivity between primary and metastatic tumors was not significant. All tumors contained TILs. TIM-3 was expressed in all TET. Moderate and high TIM-3 expression was observed in 73% of cases. All TET expressed GITR. Moderate and high expression was observed less frequently (52% of cases). Expression of ICOS and CTLA-4 was observed in 91% of cases. Their frequency of moderate and high expression was comparable to those of GITR. In cases where CD3+ TILs (IHC2 or 3 compared to IHC1) were moderately or highly present, then the OS was increased (mOS 80 months versus 43 months). In contrast, the presence of CD8+ TILs did not impact the OS. According to the authors, PD-L1 expression could be considered as a surrogate marker for an “inflamed” TME, which results in an effective host response to the tumor and prolonged survival because of native anti-tumor immunity [11].

Inhibitors of PD-1/PD-L1, such as pembrolizumab and nivolumab, act by decreasing the immune escape of tumor cells. Rajan et al. studied the treatment of advanced-stage thymomas by administrating the PD-L1 antibody avelumab. The study demonstrated a contrast of immune cell subgroups between patients that responded to treatment compared to those that did not. More specifically, patients with a response to treatment had decreased proportions of regulatory dendritic cells, T cells, B cells, and natural killer cells prior to treatment [26].

Yokoyama et al. analyzed 44 cases of thymomas. They demonstrated that high PD-L1 expression was statistically associated with advanced Masaoka stages III/IV and WHO histological types (type B2 or B3 thymoma). Disease-free survival after complete resection in high PD-L1 expression was significantly worse than in low PD-L1 expression, although there was no significant difference in OS. According to the authors, high PD-L1 expression was qualified as an independent risk factor for recurrence [22].

Moderate to high TIM-3 expression was found in the majority of TET; however, no association with high PD-L1 expression was observed. TIM-3 could be a promising therapeutic target because of its synergistic action together with anti-PD-1/PD-L1 blockade. This combined action could be the basis for future development of targeted therapies [29]. In addition, other potential targets that demonstrated moderate to high expression in this cohort are CTLA4 (52%), GITR (52%), ICOS (48%), and CD137 (54%). Increased expression of CD137 was commonly combined with a high presence of CD8+ TILs. In particular, CD137 inhibition represents an interesting field of research. Its combination with PD-1 blockade showed encouraging results in patients suffering from melanoma (response rate 50%, without any difference in terms of PD-L1 expression in the tumor) [30].

Hou et al. analyzed the TME of 21 TET by using RNA sequencing and whole exome sequencing in order to identify potential prognostic factors [9]. The immune score of thymic carcinomas presented no significant difference compared to thymomas. In addition, type A thymoma and thymic carcinoma had immune scores that were significantly lower than those of other subtypes. In contrast, thymic carcinomas had a stromal score that was significantly higher compared to thymomas. The extent of immune cell infiltration was higher in thymoma in comparison with thymic carcinoma. The predominant types of cells were those with antitumor function (such as activated CD+8 T cells, activated dendritic cells, etc.). Among cytokines involved in immune response, TNF Receptor Superfamily Member 14 (TNFRSF14) and high mobility group box 1 (HMGB1) were pro-inflammatory genes. HMGB1 showed higher expression in type A, B1, and B2 of thymomas compared to thymic carcinomas. In the case of the lower expression extent of HMGB1, the OS was significantly worse. Several studies have demonstrated that higher immune/stromal scores resulted in a better prognosis for various types of human solid malignant tumors, such as hepatocellular carcinoma, pancreatic cancer, melanoma, and lung cancer [31]. However, in the particular case of TET, stromal scores were not correlated with the immune scores, and a lower level of stromal score was found in thymoma than in thymic carcinoma. The fundamental reason is to a great degree unknown, but a possible explication could be the complexity of thymic TME, especially the immune environment, and even more complicated parameters might need to be taken into account before considering the administration of immunotherapy agents in TET.

HMGB1 is secreted by damaged or necrotic cells and acts as a damage-associated molecular sign that alerts and activates the innate immune system. Activated immune cells and endothelial cells also release HMGB1, resulting in the discharge of supplementary pro-inflammatory messengers. The capacity of HMGB1 to react to cellular stress signals and preserve lasting inflammatory process may prevent cancerogenesis and tumor growth [32].

In the study of Yang et al., 29 types of immune factors in the TME were identified, including dendritic cells (DC), antigen-presenting cells (APC) co-inhibition, APC co-stimulation, B cells, CC chemokine receptors (CCR), CD8+ T cells, checkpoint, cytolytic activity, HLA, inflammatory cells, macrophages, mast cells, MHC class I, neutrophils, natural killer (NK) cells, para-inflammation, T-cell co-inhibition, T-cell co-stimulation, T helper cells (T follicular helper cells, type 1 T helper cells, type 2 T helper cells), TILs, regulatory T cells (Treg), type I interferon (IFN) response, and type II IFN response. Specimens from thymomas were classified into different immunotypes (A and B) according to immune cell infiltration. The immunotype A group contained a higher proportion of immune factors, such as neutrophils, Th2 cells, TILs, iDCs, and CD8+ T cells, than did the immunotype B group. The immunotype B group consisted of more immune factors, such as macrophages, NK cells, Treg, Type II IFN response, and aDCs, compared to the immunotype A group. Furthermore, the survival analysis revealed that the immunotype A group resulted in significantly better survival [33].

It is also noteworthy that treatments targeting the immune cells in the TME and aiming to re-initiate the innate immune system (macrophage therapies, DC therapies, etc.) are not the subject of research in the field of TET, in contrast to other malignancies [2].

### 3.3. Tumor Mutational Burden (TMB) and Immune Infiltration

Tumor mutational burden (TMB) and immune infiltration are critical elements of cancerogenesis [34]. TMB is defined as the amount of gene mutation that occurs in the genome of a cancer cell. Consequently, TMB can be a marker of the capacity of tumors to produce new antigens [34]. An elevated TMB is responsible for the production of more antigens, and, therefore, the capacity of the tumor to provoke an immune response is increased. As a result, the T cell response and anti-tumor response is stronger, and, thus, the tumor is more suitable for immunotherapy treatment, and the benefit from ICI treatment is more probable [35,36]. TMB could eventually predict the effect of immunotherapy. In the study of Wang et al., the TET samples were classified according to the median value into low- and high-TMB groups. It was demonstrated that there is a significant association between TMB and more advanced clinical stage (Masaoka-Koga), more advanced pathological type, and increased patient age. In contrast, no notable difference in terms of PD-L1 expression was observed between the two groups. The long-term prognosis of high-TMB patients was significantly lower compared to the low-TMB group. The low- and high-TMB groups also presented significant dissimilarities in terms of infiltration levels of plasma cells, activated NK cells, macrophages, resting mast cells, activated mast cells, neutrophils, T cells CD4 naive, regulatory T cells, naive B cells, and B cell memory [37]. Increased CD8+ and CD20+ in tumor islets and in stroma that surrounds cancer cells, decreased CD204+ in tumor islets, high Forkhead box P3+ (FOXP3+), increased presence of CD8+/CD204+, and high CD20+/CD204+ in stroma are considered predictive factors of tumor recurrence in the study of Sato et al., who investigated the evolution of thymic carcinomas [38].

### 3.4. Tumor-Associated Macrophages (TAMs)

As already mentioned, the TME contains malignant cells, immune cells, mesenchymal cells, and ECM with different functions, and plays a crucial role in the different stages of tumor growth, invasion, and metastasis. Tumor-associated macrophages (TAMs) are a subtype of immune cells that infiltrate tumor tissues, and, thus, they are abundantly present in the TME. In the study of Wang et al., the cluster with a high TMB had increased macrophage presence [34]. M1 is considered to be capable of eliminating bacteria and tumor cells, and can release many different proinflammatory cytokines. In contrast, M2 inhibits the immune response and promotes angiogenesis and tissue remodeling [39]. Thymic carcinoma hosts an increased proportion of CD163+ compared to thymomas. It is also demonstrated that TAMs can secrete different factors, such as epidermal growth factor (EGF) and transforming growth factor beta 1 (TGF-B1), that promote cell proliferation and survival [40]. Consequently, TAMs are necessary for the growth of many tumor types. In addition, TAMs have a major role in tumor invasiveness because they stimulate the overexpression of proteolytic enzymes, plasmin, matrix metalloproteinases (MMPs), urokinase-type plasminogen activator (uPA), and their receptors. As a result, tumor cells are capable of degrading the extracellular matrix, destroying the basement membrane, and invading the surrounding tissue [41]. Other studies have demonstrated that the activation of the phosphatidylinositol-3-kinase/mammalian target of rapamycin (PI3K/mTOR) signal transduction pathway can result in M2 polarization of macrophages, while inhibition of the same pathway leads to M1 polarization [42,43]. Therefore, the associations among TAMs, TMB, and long-term prognosis could be the basis of further investigation.

### 3.5. The Heat Shock Protein 27 and 70 (HSP27 and 70) Expression

Heat Shock Proteins (HSP) have anti-apoptotic properties and, thus, effectuate a key action in tumor initiation, survival, and metastasis [44,45,46,47,48]. These are proteins that exist in almost all types of nucleated cells, and they are expressed in many types of malignancies, such as colon cancer, prostate cancer, hepatocellular carcinoma, lung cancer, and oral squamous cell carcinoma. The overexpression of HSP in cancer cells also accounts for resistance to chemotherapy, metastatic potential, and poor survival [44,45,46,47,48,49]. Janik et al. investigated the expression of HSP27 and 70 in 101 patients with TET [49]. HSP27 and 70 were both expressed in endothelial cells, fibroblasts, adipocytes, and macrophages, but were absent in lymphocytes. The strongest HSP70 expression in cells of the TME was observed in macrophages, especially in type AB thymomas. In contrast, the lowest HSP70 expression in fibroblasts was found in type B3 thymomas, and it was significantly weaker compared to fibroblasts of the TME in type AB thymomas. HSP70 expression in the cytoplasm of TET was higher than in stromal cells. However, the expression of HSP27 in stromal cells was significantly higher compared to HSP70.

The main action of extracellular HSP27 and 70 is to provide danger signals, resulting in a long-term inflammatory TME that promotes cancer progression and invasion. These proteins could be a potential target for the development of anticancer therapies. More specifically, investigation of HSP90 inhibitors is ongoing, both in the preclinical and clinical setting, with promising results (especially when combined with other antineoplastic regimens), even if the detailed modes of their action are largely unknown. However, it must be taken into consideration that administration of HSP inhibitors showed substantial adverse effects. According to researchers, the toxicity of HSP inhibitors is the result of their targeting the basic and fundamental expression of HSP in the rest of the organism. They suggest that a combination of HSP inhibitors can decrease drug resistance and toxicity [37,50].

### 3.6. Fibronectin

Fibronectin (FN) is an attractive target of the stroma. It is a protein that is profusely expressed on blood neo-vessels of cancer tissues [51]. FN is considered to be undetectable in adult healthy tissues, apart from specific conditions, such as tissue remodeling and repair, fibrosis, and cell migration [52,53]. The extra-domain B fibronectin (ED-B FN) is abundantly expressed in TET, with a predominant expression in the stromal cells of the thymoma microenvironment [53]. Petrini et al. selected 11 patients with recurrent TET enrolled in a prospective theragnostic phase I/II trials with radretumab, an ED-B FN specific recombinant human antibody. Radretumab radioimmunotherapy (R-RIT) was administered to eligible patients. High ED-B FN expression of the target in the peripherical microenvironment was confirmed by immunohistochemistry. The target was more abundantly expressed in B3 thymomas. According to the authors, thymomas induce stromal cells to shift FN production to the ED-B subtype, which is a crucial step for tumor progression and spread. However, despite the demonstration of the target expression, the R-RIT proved to be inefficacious in terms of objective responses. As the authors advocate, one of the major obstacles in developing a TME-specific treatment strategy is the presence of tumor heterogeneity [53].

### 3.7. SOX (SRY-Related High-Mobility Group Box) 9

SOX9 is an HMG-box transcription factor in the SOX family of proteins, and it plays a key role in tumor development and progression, including tumor initiation, TME regulation, metastasis, and drug resistance [54,55,56,57]. Yuan et al. used the immunohistochemistry method to demonstrate that SOX9 was profusely expressed in the epithelial thymic cells, especially in the epithelial cells of Hassall’s corpuscles. Moreover, SOX9 was largely expressed in the nuclei of TET tumor cells, and, according to the authors, it could be a diagnostic marker for thymomas [54]. Additionally, high expression of SOX9 was observed in 70% of type A, 50% of type AB, 22.22% of type B2 thymomas, and 45% of thymic carcinomas cases. Moreover, it was revealed that patients with increased SOX9 expression had shorter mOS. The authors advocate that SOX9 expression is correlated with the histological type of thymomas, and, consequently, it could be a dismal prognostic marker for thymomas. In conclusion, the authors suggest that SOX9 expression might be associated with an immune suppressive microenvironment of thymomas.

## 4. Discussion

TET is a group of rare neoplasms. However, they are the more frequent tumors of the prevascular mediastinum [7]. They are characterized by histological heterogeneity. Their rarity and heterogeneity are the main obstacles in the complete understanding of their molecular basis because the conduction of large-scale studies is difficult. For the same reason, the available body of literature consists of mainly retrospective studies that contain variable proportions of each histological type, requiring caution while interpreting the provided results. This review paper demonstrated that the understanding of the TME of TET has many clinical implications. The development of novel targeted therapies could lead to a paradigm shift in the treatment of advanced TET, for which cisplatin-based combination chemotherapy remains the treatment of first choice, without standard second-line treatment. Unfortunately, as already demonstrated, the complex function of the thymus and its key role in immunity inhibits the routine use of ICI.

**Figure 1:**
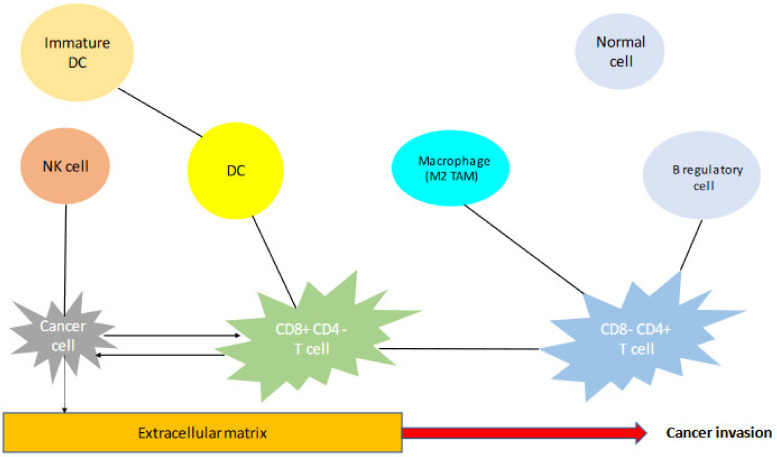
The main elements of the TME and their interaction.

The TME is a complex structure containing many different elements that are in a continuous dynamic state. The main elements of the TME are demonstrated in a simple presentation in Figure 1. Every element of the TME has a unique function that is essential in tumor genesis, survival, and spread. For example, the fibroblasts of the TME are called cancer-associated fibroblasts, and they imitate the function of normal cells, which means they produce and install ECM [58]. The endothelial cells, by secreting their growth factors, construct the network of neo-vessels, which are essential for the delivery of oxygen to the tumor [59]. As already mentioned, the immune cells can have a favorable effect in tumor genesis and progression, while others can have anti-tumoral functions. This dynamic state and equilibrium in the TME could, however, twist the balance to one or the other side, resulting, therefore, in tumor evolution or suppression [27]. From all the above, it is obvious that the TME of TET represents a wide field, where more research is warranted in order to draw pertinent conclusions concerning the predictive role of each element that is described. A summary of different prognostic factors, as identified by this review, is demonstrated in Table 1. As already mentioned, there is still some controversy concerning some of these factors, and, therefore, their real predictive role is questioned. For all the above reasons, the development of national and international databases containing not only clinical, but also molecular data could enhance the available body of literature and expand knowledge and understanding of this complex group of tumors.

## 5. Conclusions

Recently, cancer treatment started shifting towards molecular-targeted therapies. Nevertheless, the rarity and histological heterogeneity of TET constitute an obstacle in the development of new treatments, and, therefore, cytotoxic chemotherapy remains the systemic treatment of choice for advanced and unresectable tumors. Cancer progression is a complex process, during which an interaction between the tumor and its surrounding environment takes place. In-depth research and understanding of the unique characteristics of TME of TET will guide the development of new therapies. Cellular therapies may represent a fascinating field of research, because one of the main ways that cancers spread is through reprogramming innate immune cells to stimulate tumor growth and survival. Consequently, reinitiating the innate immune system is a potentially important approach to improve patient outcomes. However, the development of pertinent biomarkers will allow the selection of patients who could benefit from the administration of immunotherapy agents.

## Figures and Tables

**Table 1:** Elements of the tumor microenvironment of thymic epithelial tumors associated with favorable and dismal prognosis.

**Favorable Prognosis**
High PD-L1 expression on tumor cells (controversial) [11]
Moderate and high levels of CD3+ TILs (IHC2 or 3 vs IHC1) [11]
Higher proportion of neutrophils, Th2 cells, TILs, iDCs, CD8+ T cells [31]
**Dismal Prognosis**
High PD-L1 expression on tumor cells (controversial) [16]
Lower expression level of HMGB1 [9]
Higher proportion of immune macrophages, NK cells, Treg, Type II IFN response, and aDCs [31]
High tumor mutational burden [35]
Tumor-associated macrophages (further investigation is needed) [38,39,40,41,42]
HSP27 and 70 expression [42,43,44,45,46,47]
High SOX9 expression [52]

PD-L1: programmed death ligand 1, TILs: tumor infiltrating lymphocytes, Th2 cells: T helper 2 cells, iDCs: immature dendritic cells, HMGB1: high mobility group box 1, NK cells: natural killer cells, Treg: T regulatory cells, aDCs: activated dendritic cells, HSP27 and 70: Heat Shock Protein 27 and 70, SOX9: SRY-related high-mobility group box 9.

## References

[1] Spill F., Reynolds D.S., Kamm R.D., Zaman M.H. (2016). Impact of the physical microenvironment on tumor progression and metastasis. Curr. Opin. Biotechnol..

[2] Hinshaw D.C., Shevde L.A. (2019). The tumor microenvironment innately modulates cancer progression. Cancer Res..

[3] Alfarouk K.O., Muddathir A.K., Shayoub M.E.A. (2011). Tumor Acidity as Evolutionary Spite. Cancers.

[4] Boedtkjer E., Pedersen S.F. (2020). The Acidic Tumor Microenvironment as a Driver of Cancer. Annu. Rev. Physiol..

[5] Ramishetti S., Huang L. (2012). Intelligent design of multifunctional lipid-coated nanoparticle platforms for cancer therapy. Ther. Deliv..

[6] Thapa P., Farber D.L. (2019). The Role of the Thymus in the Immune Response. Thorac. Surg. Clin..

[7] Wright C.D. (2008). Management of thymomas. Crit Rev. Oncol. Hematol..

[8] Meng F., Wang S., Zhang J., Yan Y., Wang C., Yang C., Guan Z., Wang C. (2019). Alteration in gene expression profiles of thymoma: Genetic differences and potential novel targets. Thorac. Cancer.

[9] Hou X., Lin S., Liu Y., Wang K., Yu Z., Jia J., Yu J., Zheng W., Bai J., Chang L. (2022). Analysis of the tumor microen-vironment and mutation burden identifies prognostic features in thymic epithelial tumors. Am. J. Cancer Res..

[10] Baethge C., Goldbeck-Wood S., Mertens S. (2019). SANRA—A scale for the quality assessment of narrative review articles. Res. Integr. Peer Rev..

[11] Arbour K.C., Naidoo J., Steele K.E., Ni A., Moreira A.L., Rekhtman N., Robbins P.B., Karakunnel J., Rimner A., Huang J. (2017). Expression of PD-L1 and other immunotherapeutic targets in thymic epithelial tumors. PLoS ONE.

[12] Giaccone G., Thompson J., McGuire C., Manning M., Kallakury B., Chahine J.J., Subramaniam D.S., Liu S.V., Gibney G.T., Kim C. (2017). Pembrolizumab in patients with recurrent thymic carcinoma: Results of a phase II study. J. Clin. Oncol..

[13] Giaccone G., Kim C., Thompson J., McGuire C., Kallakury B., Chahine J.J., Manning M., Mogg R., Blumenschein W.M., Tan M.T. (2018). Pembrolizumab in patients with thymic carcinoma: A single-arm, single-centre, phase 2 study. Lancet Oncol..

[14] Tateo V., Manuzzi L., De Giglio A., Parisi C., Lamberti G., Campana D., Pantaleo M. (2020). Immunobiology of Thymic Epithelial Tumors: Implications for Immunotherapy with Immune Checkpoint Inhibitors. Int. J. Mol. Sci..

[15] Bedekovics J., Beke L., Mokanszki A., Szilagyi S., Mehes G. (2020). Programmed Death-ligand 1 (PD-L1) Expression in Thymic Epithelial Tumors. Appl. Immunohistochem. Mol. Morphol..

[16] Padda S.K., Riess J.W., Schwartz E.J., Tian L., Kohrt H.E., Neal J.W., West R.B., Wakelee H.A. (2015). Diffuse High Intensity PD–L1 Staining in Thymic Epithelial Tumors. J. Thorac. Oncol..

[17] Katsuya Y., Fujita Y., Horinouchi H., Ohe Y., Watanabe S.-I., Tsuta K. (2015). Immunohistochemical status of PD-L1 in thymoma and thymic carcinoma. Lung Cancer.

[18] Yokoyama S., Miyoshi H., Nakashima K., Shimono J., Hashiguchi T., Mitsuoka M., Takamori S., Akagi Y., Ohshima K. (2016). Prognostic Value of Programmed Death Ligand 1 and Programmed Death 1 Expression in Thymic Carcinoma. Clin. Cancer Res..

[19] Su Y., Ou Y., Chen Y., Ma X. (2022). Construction of immune-related LncRNAs classifier to predict prognosis and immunotherapy response in thymic epithelial tumors. Biosci. Rep..

[20] Katsuya Y., Horinouchi H., Seto T., Umemura S., Hosomi Y., Satouchi M., Nishio M., Kozuki T., Hida T., Sukigara T. (2019). Single-arm, multicentre, phase II trial of nivolumab for unresectable or recurrent thymic carcinoma: PRIMER study. Eur. J. Cancer.

[21] Cho J., Kim H.S., Ku B.M., Choi Y.-L., Cristescu R., Han J., Sun J.-M., Lee S.-H., Ahn J.S., Park K. (2019). Pembrolizumab for Patients With Refractory or Relapsed Thymic Epithelial Tumor: An Open-Label Phase II Trial. J. Clin. Oncol..

[22] Yokoyama S., Miyoshi H., Nishi T., Hashiguchi T., Mitsuoka M., Takamori S., Akagi Y., Kakuma T., Ohshima K. (2016). Clinicopathologic and Prognostic Implications of Programmed Death Ligand 1 Expression in Thymoma. Ann. Thorac. Surg..

[23] Chen H.F., Wu L.X., Li X.F., Zhu Y.C., Pan W.W., Wang W.X., Xu C.W., Huang J.H., Wu M.H., Du K.Q. (2020). PD-L1 Ex-pression Level in Different Thymoma Stages and Thymic Carcinoma: A Meta-Analysis. Tumori.

[24] Yokoyama S., Miyoshi H. (2018). Thymic tumors and immune checkpoint inhibitors. J. Thorac. Dis..

[25] Weissferdt A., Fujimoto J., Kalhor N., Rodriguez J., Bassett R., Wistuba I., Moran C. (2017). Expression of PD-1 and PD-L1 in thymic epithelial neoplasms. Mod. Pathol..

[26] Rajan A., Heery C.R., Thomas A., Mammen A.L., Perry S., O’Sullivan Coyne G., Guha U., Berman A., Szabo E., Madan R.A. (2019). Efficacy and Tolerability of Anti-programmed Death-Ligand 1 (PD-L1) Antibody (Avelumab) Treatment in Advanced Thymoma. J. Immunother. Cancer.

[27] Masaoutis C., Palamaris K., Kokkali S., Levidou G., Theocharis S. (2022). Unraveling the Immune Microenvironment of Thymic Epithelial Tumors: Implications for Autoimmunity and Treatment. Int. J. Mol. Sci..

[28] Fend F., Kirchner T., Marx A., Müller-Hermelink H.-K. (1993). B-cells in thymic epithelial tumours. Virchows Arch. B Cell Pathol. Incl. Mol. Pathol..

[29] Ngiow S.F., von Scheidt B., Akiba H., Yagita H., Teng M.W.L., Smyth M.J. (2011). Anti-TIM3 Antibody Promotes T Cell IFN-γ–Mediated Antitumor Immunity and Suppresses Established Tumors. Cancer Res..

[30] Parkhurst M., Gros A., Pasetto A., Prickett T., Crystal J.S., Robbins P., Rosenberg S.A. (2017). Isolation of T-Cell Receptors Specifically Reactive with Mutated Tumor-Associated Antigens from Tumor-Infiltrating Lymphocytes Based on CD137 Ex-pression. Clin. Cancer Res..

[31] Ros-Martínez S., Navas-Carrillo D., Alonso-Romero J.L., Orenes-Piñero E. (2020). Immunoscore: A novel prognostic tool. Association with clinical outcome, response to treatment and survival in several malignancies. Crit. Rev. Clin. Lab. Sci..

[32] Kang R., Chen R., Zhang Q., Hou W., Wu S., Cao L., Huang J., Yu Y., Fan X.G., Yan Z. (2014). HMGB1 in health and disease. Mol. Asp. Med..

[33] Yang Y., Xie L., Li C., Liu L., Ye X., Han J. (2022). Prognostic Model of Eleven Genes Based on the Immune Microenvironment in Patients With Thymoma. Front. Genet..

[34] Wang Z., Xu Q., Kaul D., Ismail M., Badakhshi H. (2021). Significance of tumor mutation burden and immune infiltration in thymic epithelial tumors. Thorac. Cancer.

[35] Goodman A.M., Kato S., Bazhenova L., Patel S.P., Frampton G.M., Miller V., Stephens P.J., Daniels G.A., Kurzrock R. (2017). Tumor Mutational Burden as an Independent Predictor of Response to Immunotherapy in Diverse Cancers. Mol. Cancer Ther..

[36] Buttner R., Longshore J.W., Lopez-Rios F., Merkelbach-Bruse S., Normanno N., Rouleau E., Penault-Llorca F. (2019). Imple-menting TMB measurement in clinical practice: Considerations on assay requirements. ESMO Open.

[37] Wang X., Chen M., Zhou J., Zhang X. (2014). HSP27, 70 and 90, anti-apoptotic proteins, in clinical cancer therapy (Review). Int. J. Oncol..

[38] Sato J., Kitano S., Motoi N., Ino Y., Yamamoto N., Watanabe S., Ohe Y., Hiraoka N. (2020). CD20 + Tumor-infiltrating Immune Cells and CD204 + M2 Macrophages Are Associated with Prognosis in Thymic Carcinoma. Cancer Sci..

[39] Gordon S. (2003). Alternative activation of macrophages. Nat. Rev. Immunol..

[40] Goswami S., Sahai E., Wyckoff J.B., Cammer M., Cox D., Pixley F.J., Stanley E.R., Segall J.E., Condeelis J.S. (2005). Macrophages promote the invasion of breast carcinoma cells via a colony-stimulating factor-1/epidermal growth factor paracrine loop. Cancer Res..

[41] Migita T., Sato E., Saito K., Mizoi T., Shiiba K.-I., Matsuno S., Nagura H., Ohtani H. (1999). Differing expression of MMPs-1 and -9 and urokinase receptor between diffuse- and intestinal-type gastric carcinoma. Int. J. Cancer.

[42] Souza-Moreira L., Soares V.C., Dias S., Bozza P.T. (2019). Adipose-derived mesenchymal stromal cells modulate lipid metabolism and lipid droplet biogenesis via AKT/mTOR -PPARgamma Signalling in macrophages. Sci. Rep..

[43] Chen W., Ma T., Shen X.N., Xia X.F., Xu G.D., Bai X.L., Liang T.B. (2012). Macrophage-induced tumor angiogenesis is reg-ulated by the TSC2-mTOR pathway. Cancer Res..

[44] Lazaris A.C., Theodoropoulos G.E., Davaris P.S., Panoussopoulos D., Nakopoulou L., Kittas C., Golematis B.C. (1995). Heat shock protein 70 and HLA-DR molecules tissue expression. Dis. Colon Rectum.

[45] Glaessgen A., Jonmarker S., Lindberg A., Nilsson B., Lewensohn R., Ekman P., Valdman A., Egevad L. (2008). Heat shock proteins 27, 60 and 70 as prognostic markers of prostate cancer. Apmis.

[46] Joo M., Chi J.G., Lee H. (2005). Expressions of HSP70 and HSP27 in Hepatocellular Carcinoma. J. Korean Med Sci..

[47] Małusecka E., Krzyzowska-Gruca S., Gawrychowski J., Fiszer-Kierzkowska A., Kołosza Z., Krawczyk Z. (2008). Stress proteins HSP27 and HSP70i predict survival in non-small cell lung carcinoma. Anticancer Res..

[48] Mohtasham N., Babakoohi S., Montaser-Kouhsari L., Memar B., Salehinejad J., Rahpeyma A., Khageh-Ahmady S., Marouzi P., Firooz A., Pazoki-Toroudi H. (2011). The expression of heat shock proteins 27 and 105 in squamous cell carci-noma of the tongue and relationship with clinicopathological index. Med. Oral. Pathol. Oral. Cir. Bucal..

[49] Janik S., Schiefer A.I., Bekos C., Hacker P., Haider T., Moser J., Klepetko W., Müllauer L., Ankersmit H.J., Moser B. (2016). HSP27 and 70 expression in thymic epithelial tumors and benign thymic alterations: Diagnostic, prognostic and physiologic implications. Sci. Rep..

[50] McConnell J.R., McAlpine S.R. (2013). Heat shock proteins 27, 40, and 70 as combinational and dual therapeutic cancer targets. Bioorg. Med. Chem. Lett..

[51] Rick J.W., Chandra A., Ore C.D., Nguyen A.T., Yagnik G., Aghi M.K. (2019). Fibronectin in malignancy: Cancer-specific alterations, protumoral effects, and therapeutic implications. Semin. Oncol..

[52] Menrad A., Menssen H.D. (2005). ED-B fibronectin as a target for antibody-based cancer treatments. Expert Opin. Ther. Targets.

[53] Petrini I., Sollini M., Bartoli F., Barachini S., Montali M., Pardini E., Burzi I.S., Erba P.A. (2022). ED-B-Containing Isoform of Fibronectin in Tumor Microenvironment of Thymomas: A Target for a Theragnostic Approach. Cancers.

[54] Yuan X., Huang L., Luo W., Zhao Y., Nashan B., Yu F., Liu Y. (2021). Diagnostic and Prognostic Significances of SOX9 in Thymic Epithelial Tumor. Front. Oncol..

[55] Grimm D., Bauer J., Wise P., Krüger M., Simonsen U., Wehland M., Infanger M., Corydon T.J. (2019). The role of SOX family members in solid tumours and metastasis. Semin. Cancer Biol..

[56] Panda M., Tripathi S.K., Biswal B.K. (2021). SOX9: An emerging driving factor from cancer progression to drug resistance. Biochim. Biophys. Acta Rev. Cancer.

[57] Matheu A., Collado M., Wise C., Manterola L., Cekaite L., Tye A.J., Canamero M., Bujanda L., Schedl A., Cheah K.S. (2012). Oncogenicity of the Developmental Transcription Factor Sox9. Cancer Res..

[58] Bhowmick N.A., Neilson E.G., Moses H.L. (2004). Stromal fibroblasts in cancer initiation and progression. Nature.

[59] Carmeliet P., Jain R.K. (2000). Angiogenesis in cancer and other diseases. Nature.

